# Pylephlebitis: A Rare but Redoubtable Complication of Intra-Abdominal Infections—A Series of 15 Cases

**DOI:** 10.3390/life15101525

**Published:** 2025-09-26

**Authors:** Serban Nicolae Benea, Teodora Deaconu, Dragos Florea, Ruxandra Moroti, Gabriela Oprica, Alina Nae, Raluca Elena Patrascu, Eliza Militaru, Habip Gedik, Ilinca Savulescu-Fiedler

**Affiliations:** 1 Faculty of Medicine, Carol Davila University of Medicine and Pharmacy, 050474 Bucharest, Romania; serban.benea@umfcd.ro (S.N.B.); teodora.gadea@rez.umfcd.ro (T.D.); maria-gabriela.oprica@rez.umfcd.ro (G.O.); alina.nae@rez.umfcd.ro (A.N.); raluca.jipa@umfcd.ro (R.E.P.); eliza.manea@drd.umfcd.ro (E.M.); ilinca.savulescu@umfcd.ro (I.S.-F.); 2 National Institute for Infectious Diseases “Prof. Dr. Matei Bals”, 021105 Bucharest, Romania; 3 Department of Infectious Diseases and Clinical Microbiology, Hamidiye School of Medicine, University of Health Sciences Istanbul, 34668 Istanbul, Turkey; habip.gedik@sbu.edu.tr; 4 Department of Internal Medicine, Coltea Clinical Hospital, 030167 Bucharest, Romania

**Keywords:** pylephlebitis, suppurative portal vein thrombosis, liver abscess, intra-abdominal infection complications, sepsis

## Abstract

Pylephlebitis is the suppurative thrombosis of the portal vein system. Mainly reported as a severe complication of diverticulitis or appendicitis, it is an uncommon intra-abdominal infection: approximately 200 cases have been reported in the English literature, mostly from surgical wards. Our study aims to assess the role of an infectious disease setting in managing pylephlebitis. We reviewed medical records from 2001 to 2024 at a tertiary infectious diseases hospital and identified 15 cases. The median age was 58 years [IQR = 28], with a male-to-female ratio of 4:1. Along with portal vein thrombosis (PVT), liver abscess(es) was/were the main radiological finding (*n* = 12), representing 80% of cases. The liver abscesses appear as secondary events in the case of pylephlebitis. In seven of 15 cases, we found the primary event associated with pylephlebitis. Blood cultures were positive in eight cases, with Gram-negative aerobic bacteria being commonly isolated (*n* = 5), followed by anaerobes (*n* = 3); in half of the cases, more than one pathogen was involved. All patients received broad-spectrum antibiotics containing beta-lactams, including eight who received carbapenems. Anticoagulation therapy was used in 14 cases. Two deaths were recorded, and four patients required surgical intervention, highlighting the importance of prompt diagnosis and swift antibiotic and anticoagulant treatment.

## 1. Introduction

### 1.1. Definition

PVT (portal vein thrombosis) is the complete or partial obstruction of the portal vein or its branches by a thrombus. PVT may extend retrogradely into its afferent vessels—such as the superior mesenteric vein and splenic vein—or anterogradely into its intrahepatic branches [1,2]. PVT is a rare event in the general population, but it is more common among patients with liver cirrhosis or malignancy, especially in cases of liver or pancreatic cancers [2].

Pylephlebitis is a subcategory of PVT, also named infected PVT, suppurative PVT, septic PVT, or portal pyaemia. The portal vein drains the abdominal gastrointestinal tract, except for the lower rectum, which drains into the inferior cava vein. This explains why almost all intra-abdominal or pelvic infections can lead to pylephlebitis [3].

Pylephlebitis is a rare clinical condition. The reported incidence ranges from approximately 0.37 to 2.7 cases per 100,000 person-years [4], with a higher incidence in males [4,5,6,7]. Only 220 confirmed cases of pylephlebitis, based on culture results and imaging, have been published in the English literature between 1970 and 2022 [4].

Patients with pylephlebitis may exhibit various clinical presentations, ranging from asymptomatic cases (discovered incidentally during an imaging test) to more severe conditions, such as sepsis and multi-organ failure [4,5,7].

The therapeutic strategies (including antibiotics, anticoagulants, and/or surgery in selected cases) are not always clear due to the absence of randomised trials, a consequence of its low incidence [4,5,7].

Most studies report high mortality rates ranging from 11% to 32%, even with prompt and appropriate management [5].

### 1.2. Pathophysiology

#### 1.2.1. PVT

The three categories of factors comprising Virchow’s triad are involved in PVT pathophysiology, each contributing differently depending on the PVT aetiology: hypercoagulable status, slowed portal blood flow, and vascular endothelial injury [8].

- The hypercoagulable status is the primary determinant of PVT in patients with inherited or acquired thrombophilia. An example of acquired thrombophilia is primary myeloproliferative disorders. Another group of conditions associated with hypercoagulable status includes neoplastic diseases. In some cases, direct invasion of the portal vein (such as in hepatocellular carcinoma and cholangiocarcinoma), or tumour or lymph node compression, may favour PVT [8].
- Slowed portal blood flow. Reduced portal venous flow is a key contributor to portal vein thrombosis (PVT) in patients with liver cirrhosis, primarily due to structural distortion of the hepatic architecture. Moreover, cirrhotic patients exhibit a distinct haemostatic profile known as rebalanced haemostasis, which renders them susceptible to both bleeding and thrombotic complications [8,9]. Additionally, endotoxemia—frequently observed in cirrhosis—is associated with an increased risk of thrombotic events [8].
- Endothelial (vascular) injury. Intra-abdominal inflammatory or infectious diseases, as well as local injuries related to surgical procedures, can damage the vascular endothelium [10,11,12]. Activated neutrophils in inflamed areas promote thrombin generation and thrombi formation, a phenomenon observed regardless the cirrhotic or non-cirrhotic status [13,14,15].

The thrombi progressively obstruct the portal vein lumen, increasing pressure within the portal system, impairing anterogradely hepatic and retrogradely venous mesenteric and splenic blood flow. The resulting blood stagnation could promote anterogradely hepatic ischemia and endothelial damage, further propagating thrombosis into intrahepatic branches and retrogradely mesenteric ischemia and splenic enlargement.

Once established, thrombosis may cause partial or complete obstruction of portal circulation, subsequently leading to secondary portal hypertension. This condition leads to the development of portosystemic collateral circulation [16,17] and, in severe cases, acute liver failure [18].

#### 1.2.2. Pylephlebitis

**Figure 1:**
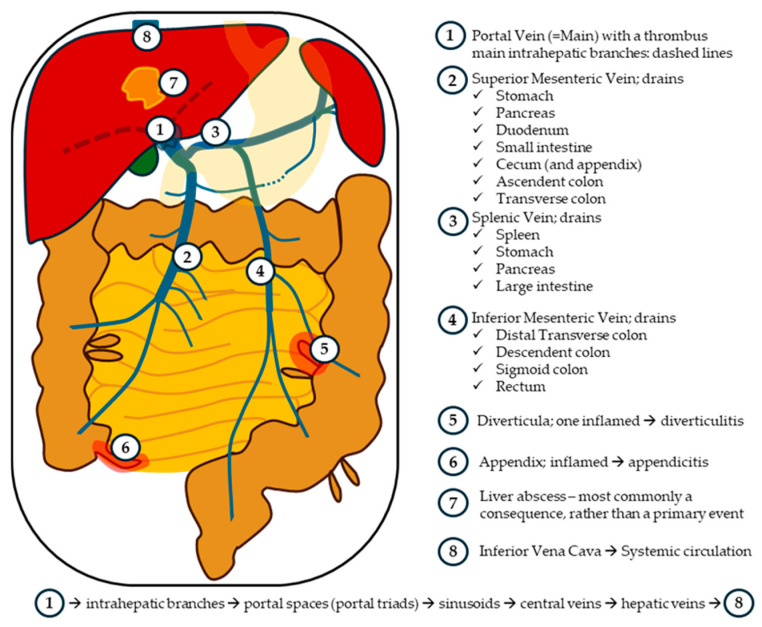
Portal Vein and Its Tributaries: A Regional Drainage Overview. Legend: Pylephlebitis, or septic thrombosis of the portal vein, may arise as a complication of various intra-abdominal infectious processes, most commonly diverticulitis and appendicitis. Other potential sources include cholecystitis, cholangitis, pancreatitis, liver abscess, colitis, prostatitis, and even postoperative intra-abdominal infections. In this illustration, appendicitis and diverticulitis are highlighted as they represent the most frequent aetiologies. Illustration by Ruxandra Moroti. All rights reserved.

This severe infectious complication of the portal venous system is primarily driven by the third component of Virchow’s triad described above (Section 1.2.1): endothelial injury of the portal vein and its branches. Endothelial injury may result in case of an intraabdominal infection, most commonly diverticulitis or appendicitis, from which bacteria invade the mesenteric venous system and subsequently the portal vein (Figure 1). The colonisation of the portal vascular endothelium by pathogens—mainly *Escherichia coli*, *Bacteroides fragilis*, and streptococci—triggers a strong inflammatory response, leading to endothelial activation, the initiation of the coagulation cascade, and the formation of septic thrombi in the portal vein system. This phenomenon could trigger a severe systemic inflammatory response and/or could lead to a haematogenous dissemination of septic emboli, ultimately contributing to sepsis with multiorgan failure. Returning to the portal septic thrombi, they could extend further into the liver, leading to the formation of hepatic abscesses, or propagate retrogradely into the mesenteric and splenic veins, potentially resulting in intestinal ischemia and splenic abscesses, respectively [19].

### 1.3. Aetiology and Diagnosis

The most common morbid conditions associated with pylephlebitis are diverticulitis (the most common nowadays) and appendicitis (the leading cause in the pre-antibiotic era); these two conditions account for nearly half of all causes in the largest cohort of 220 cases analysed by Fusaro et al. [4]. Other causes include inflammatory bowel disease, colitis, cholecystitis, pancreatitis, and hepatic abscess [3,4,5,6,7,20,21], with the last being a consequence rather than a primary event in cases of pylephlebitis [4]. Gallstones and hepatolithiasis are frequently associated with significant complications, such as acute cholecystitis, cholangitis, biliary pancreatitis, Mirizzi syndrome, and even biliary ileus. Intrahepatic stones may lead to recurrent cholangitis and chronic biliary stasis, with progressive parenchymal damage. These conditions may create a favourable environment for severe infectious complications, including pylephlebitis [22]. Pylephlebitis can also be a serious complication of certain invasive procedures in the hepato-biliary area, such as haemorrhoidal banding, gastric banding, CT-guided liver biopsy, or even laparoscopic surgery [23,24,25,26]. Notably, over 40% of patients had a hypercoagulable condition (such as malignancy, thrombophilia, or HIV infection) [4].

Pylephlebitis has a low incidence and nonspecific symptoms and may be misleading due to its overlap with other intra-abdominal or systemic infections, including sepsis. Therefore, diagnosis is difficult and often delayed when practitioners do not initially consider this condition. Currently, there are no standardised criteria for pylephlebitis. The most common symptoms include fever and chills (which can be persistent, for example, in the case of hepatic abscess), abdominal pain (either diffuse or localised in the right upper quadrant), nausea, vomiting, diarrhoea, and weight loss. If jaundice is present, it may indicate significant hepatic involvement [3,4,5,6,7].

Laboratory tests may reveal a nonspecific inflammatory response, with leucocytosis and elevated inflammatory markers, such as high levels of C-reactive protein (CRP) and fibrinogen. Liver function tests, including levels of alkaline phosphatase, AST, ALT, and gamma-glutamyl transferase (GGT), can be abnormal in most patients, with higher values present when hepatic involvement exists [4,5]. Blood cultures are crucial for identifying the causative germs. Common microorganisms involved in pylephlebitis include Gram-negative bacteria—such as *Escherichia coli*, *Bacteroides* species, and *Klebsiella pneumoniae*—as well as Gram-positive organisms, notably *Streptococcus* species. Fusaro et al. reported that corroborating positive cultures from blood and/or other appropriate sites could establish a diagnosis in approximately 70% of patients [4]. However, the sensitivity of blood cultures alone generally reaches a maximum of 60%. Other studies have documented bacteraemia rates ranging from 42% to 62.1% [3,5,7,27,28].

In case of intra-abdominal infection, imaging is essential. It provides direct visualisation of the portal vein and any associated abnormalities. Doppler-enhanced ultrasound is a non-invasive technique widely available in most clinical settings and can detect thrombus formation while assessing blood flow. Currently, computed tomography (CT) scan is considered the gold standard for confirming diagnosis. It reveals PVT, its extent, and affected branches. A contrast-enhanced CT scan is more advantageous because it can locate the source of infection, such as bowel thickening, abscesses, or more subtle changes like diverticulitis or appendicitis [3]. Magnetic resonance imaging, angiography, endoscopic ultrasound, and positron emission tomography are currently limited in use [4].

Overall, the diagnosis involves two steps. First, clinicians need to consider the possibility of PVT when an intra-abdominal infection is present. Second, they must order imaging studies that can confirm the presence of PVT. An additional important step is to distinguish between cirrhotic and non-cirrhotic PVT, because the clinical course and treatment could differ.

### 1.4. Evolution and Prognosis

The most critical determinants of evolution are the diagnosis time and rapid intervention.

In pylephlebitis, the most severe complications are sepsis, metastatic abscesses (including hepatic abscesses), multiorgan failure, and small bowel ischaemia. Sepsis is the most common complication and the leading cause of death. If sepsis occurs, it leads to a 17-fold increase in mortality [7].

Patients with unresolved acute PVT may progress to chronic PVT, which leads to the development of collateral hepatoportal circulation that bypasses the obstruction (portal cavernoma) [5,17,29]. Chronic PVT can cause complications related to portal hypertension. A review by Choudhry et al. reported an overall complication rate of 20% [5].

In contrast to classical PVT in non-infectious contexts, where ischaemic hepatitis, even acute liver failure and gastrointestinal bleeding are associated [17], in pylephlebitis, these complications are rarely described [4,5,7].

### 1.5. The Aim of Our Study

Our main objective was to highlight the particularities in managing patients diagnosed with pylephlebitis within the clinical setting of a monodisciplinary infectious disease hospital, considering that most of the cases reported in the literature are related to pathologies requiring surgery.

## 2. Materials and Methods

We conducted a retrospective observational study. The research was performed at the National Institute for Infectious Diseases “Prof. Dr. Matei Balș”, a tertiary care hospital in Bucharest. The study team reviewed the medical records of patients admitted to our hospital from 1 January 2001 to 31 December 2024.

We searched the hospital’s medical database for diagnoses containing terms such as “infectious portal vein thrombosis”, “septic portal vein thrombosis”, “pylephlebitis”, “suppurative portal vein thrombosis”, “portal pyaemia”, and “portal vein thrombosis”. The study team reviewed each medical record of PVT to determine whether PVT could be related to an intra-abdominal infection or if it was secondary to advanced cirrhosis, liver cancer, other malignancies, or another non-infectious condition that could precipitate a PVT (such as thrombophilia but without infection).

The inclusion criteria were the diagnosis of pylephlebitis. We defined pylephlebitis as the presence of PVT (detected by computed tomography and/or abdominal ultrasonography) plus evidence of a confirmed or suspected intra-abdominal and/or systemic infection (clinical signs of a systemic inflammatory response and/or positive blood cultures).

The exclusion criteria included hepatic transplant, liver cancer, or other intra-abdominal malignancies if they could be the primary cause of a PVT.

Patient data were collected from medical records, including complete clinical progress and treatment documentation. For each patient, potential underlying pathologies and conditions that could have contributed to the development of pylephlebitis, such as intra-abdominal infections or recent surgical procedures, were analysed. Information regarding the presence of fever and other symptoms at admission, as well as the results of imaging investigations that aided in diagnosis and identification of the affected branches of the portal vein, was also collected. Imaging investigations included abdominal ultrasound (Affinity 70, Royal Philips, Eindhoven, The Netherlands) and abdominal computed tomography (Somatom Definition AS, Siemens Healthcare GmbH, Munich, Germany). The biological parameters evaluated included haematological analyses, inflammatory markers, liver and kidney function, and microbiological cultures for pathogen identification. Blood cultures (Bactalert, Biomerieux, Lyon, France) were obtained whenever signs or symptoms of severe infection were present.

Details of treatment, including the antibiotic regimens, anticoagulant therapy, and any surgical interventions, were documented.

Complications during hospitalisation and patient outcomes, including deaths, were also recorded.

Data Analysis: This descriptive analysis presents continuous variables as means or medians, along with interquartile ranges (IQR), depending on the data distribution. Categorical variables are expressed as frequencies and percentages.

Ethical Considerations: The study received approval from the Ethics Committee of the National Institute of Infectious Diseases “Prof. Dr. Matei Balș” (No C01306/5 February 2025). Our work complied with all relevant regulations regarding the protection of personal data and patient confidentiality. The information included was anonymised, and the collected data was used solely for research purposes, without affecting patient care or rights.

## 3. Results

We identified 15 patients admitted to our institute during the study period (January 2001–December 2024). Between 2001 and 2006, no patients were identified.

### 3.1. Demographics and Risk Factors

- Out of 15 patients, 12 were men, with a male-to-female ratio of 4:1. The age at diagnosis had a median of 58 years [IQR: 28].
- The comorbidities of each patient were listed in Table 1. As PVT known risk factors, five patients reported alcohol abuse, one had thrombophilia (without being on anticoagulation treatment), one had an antiphospholipid syndrome, and one was diagnosed during admission with a pancreatic head tumour. The team carefully reviewed this case. It was finally included in the study as it was a small mass, localised in the pancreatic head, without local involvement of the lymph nodes, or criteria for local invasion (particularly in the local veins), and it was associated with a liver abscess.

### 3.2. Diagnosis

#### 3.2.1. Clinical Features

Clinical symptoms varied, but fever was observed in all 15 patients (100%).

Abdominal pain was reported in eight patients (53.3%), localised to the upper abdomen—specifically the epigastric region and right hypochondrium in four cases or being diffuse in three cases (see Table 1). In two cases, the pain was described as intermittent or colicky in nature.

Other symptoms, such as nausea/vomiting (*n* = 4), weight loss (*n* = 2), diarrhoea (*n* = 3), and jaundice (*n* = 1), were less common. In two cases, patients experienced confusion and loss of consciousness as a consequence of severe systemic complications (sepsis).

#### 3.2.2. Laboratory Nonspecific Tests

Biological parameters indicated systemic inflammation. Elevated inflammatory markers were common. CRP was measured in 11 patients, all of whom had abnormal levels, and six had levels higher than 100 mg/L. The median CRP level was 107 mg/L IQR = 110.3 [161 − 50.7], the reference value being <5 mg/dL. Fibrinogen levels were recorded for all patients, with nine exhibiting abnormal levels exceeding 400 mg/dL. The median fibrinogen level was 491 mg/dL, IQR: 267 [641 − 374], the reference range being 200–400 mg/dL D-dimer levels were measured in six out of the fifteen patients included in the study. All six patients showed positive results. Among them, two underwent qualitative analysis, which confirmed the presence of fibrinogen degradation products. The remaining four had quantitative assessments, revealing D-dimer levels with a mean value approximately 15 times higher than the normal reference range and a median value 10 times above normal. Procalcitonin levels were measured in ten patients: all had increased values (>0.05 ng/mL), and values higher than five ng/mL were observed in four.

Leucocytosis was observed, with a median leukocyte count of 12,290/mm^3^, IQR = 5440 [15,840 − 10,400]. Platelets levels varied, with a median of 304,000/mm^3^, IQR = 212,000 [409,000 − 197,000]. Only three patients had platelets count below 150,000 cells/mm^3^. All but one presented anaemia: the Haemoglobin level had a median of 10.4 mg/dL (minimum of 7.3, maximum of 13.4), a mean of 10.35 mg/dL, and an IQR of 4.2 [12.5 − 8.3].

Impairment of hepatic function was indicated by elevated liver enzymes: AST (median: 50 U/L, IQR = 66 [95 − 29]), reference range: 28–50 U/L, ALT (median: 63 U/L, IQR = 129 [157 − 28]), reference range: 24–45 U/L and GGT (median: 236 U/L, IQR = 147 [295 − 148]), reference range: 15–73 U/L. Bilirubin levels were slightly modified, with a median of 1 mg/dL and an IQR = 0.6 [1.4 − 0.8], reference range: 0.2–1 mg/dL.

#### 3.2.3. Microbiology

Out of the 15 patients who underwent blood cultures, eight had positive results, with various pathogens identified. The most isolated bacteria were *Klebsiella pneumoniae* (4 cases, 26.6%), followed by *Bacteroides fragilis* (3 cases, 20%), *Escherichia coli* (2 cases, 13.3%). *Enterococcus avium*, *Pseudomonas aeruginosa* and *Clostridium symbiosum*, were each identified in one case (6.7%). Four patients with positive blood cultures had two germs isolated simultaneously: *Klebsiella pneumoniae* and *Escherichia coli* (in one patient), *Klebsiella pneumoniae* and *Enterococcus avium* (in one patient), *Klebsiella pneumoniae* and *Pseudomonas aeruginosa* (in one patient), and *Bacteroides fragilis* and *Clostridium symbiosum* (in one patient).

Antibiotic susceptibility testing of the bacterial isolates was performed in all cases, and the strains were multi-susceptible in seven of the eight cases. In case #12, there was recovered from blood culture an ESBL producer *E coli* strain.

#### 3.2.4. Imaging

**Figure 2:**
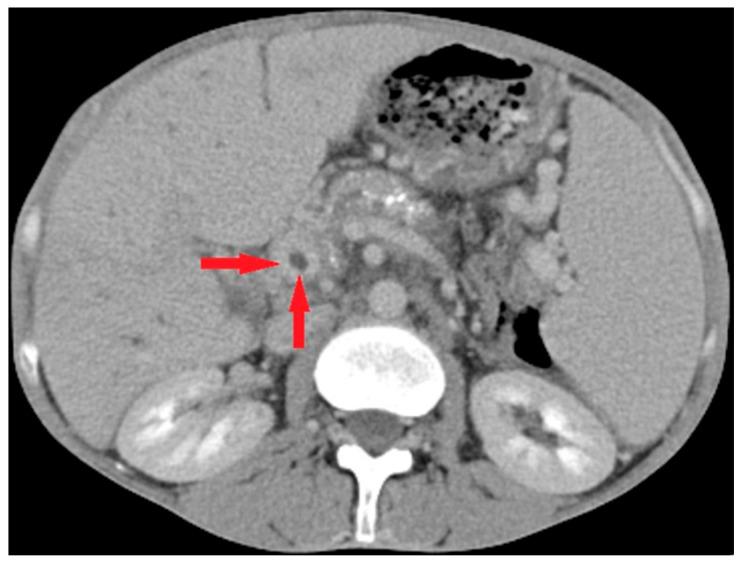
Abdominal CT scan in case of pylephlebitis. Legend: Suppurative portal vein thrombosis on CT scan seen as a central filling defect (red arrows); the illustration corresponds with case #11 described in Table 1 and Table 2.

Imaging played a crucial role in diagnosing pylephlebitis, with computed tomography remaining the primary test for identifying this condition. Among the 14 patients evaluated by computed tomography (CT), 13 had PVT (Figure 2); in two cases (one with a ‘negative’ CT and one who did not undergo a CT scan examination), the diagnosis of PVT was based solely on ultrasound examination.

Seven out of 15 patients had a probable source of infection visible on imaging tests: two patients had cholecystitis, two had diverticulitis (one of whom also had pancreatitis and the other an intra-abdominal abscess). Splenic involvement was identified in four patients (three were described as abscesses and the fourth as infarction). In one case, alongside splenic and liver abscesses, prostatic abscesses were also described (Table 1).

By combining the two imaging methods (CT and ultrasound examination), liver abscesses were identified in 12 of 15 patients (80%), of which at least seven were described as multiple; for the other eight, this information was not available.

Other findings included hepatosplenomegaly in four cases, a pancreatic head tumour without invasion in one case, and a portal cavernoma in the hepatic hilum in one case.

The radiological findings are summarised in Table 1, including the anatomical part of the portal system affected by thrombosis.

### 3.3. Treatment

#### 3.3.1. Antibiotic Therapy

In the analysed cohort, antibiotic therapy was mainly administered in combinations, with most patients receiving multiple antibiotics. Carbapenems were the most used, followed by other beta-lactams with or without beta-lactamase inhibitors, glycopeptides, fluoroquinolones, polymyxins, and oxazolidinones. Tetracyclines and aminoglycosides were used in isolated cases. Metronidazole was used in the case of *B. fragilis* bacteraemia.

The median duration of antibiotic treatment was 29 days. After excluding patients without hepatic abscess and the four patients who required surgical interventions and were transferred to another hospital, we calculated a median treatment duration of 38 days.

The treatment regimens and their duration are detailed in Table 2.

#### 3.3.2. Anticoagulant Therapy

Anticoagulant therapy, administered to 14 out of 15 patients, was essential in managing pylephlebitis. Most received low-molecular-weight heparin (LMWH), while five patients were treated with vitamin K antagonists (VKA) or direct oral anticoagulants (DOACs). Only one patient did not receive anticoagulant treatment.

#### 3.3.3. Surgery

Surgical interventions were required in four cases, all involving severe complications such as multiple liver abscesses (two cases), intra-abdominal abscesses related to diverticulitis (one case) and in one case of acute cholecystitis accompanied by sepsis (see Table 2, patients #6, #7, #8 and #14. Two patients were transferred back to our clinic after surgery and successfully continued antibiotic treatment, but no follow-up data were available for the other two.

### 3.4. Outcome

Two of the four patients who needed surgery were lost to follow-up after surgery. The remaining two returned and completed the antibiotic treatment. Two deaths occurred, resulting from multiple organ failure associated with sepsis. Five patients had data regarding follow-up and long-term sequelae reported: one had hepatic left lobe atrophy (described by CT), another had hepatic fibrosis in the affected hepatic zone, and the third had already developed a portal cavernoma and experienced an episode of acute pancreatitis two months after discharge from the hospital. The fourth and fifth patients who returned for follow-up showed no reported sequelae.

## 4. Discussion

### 4.1. Infectious Source of Pylephlebitis

In our study group, seven of 15 patients had a probable source of infection: two had diverticulitis (one of whom also had pancreatitis and the other an intra-abdominal abscess), two had cholecystitis, and four had splenic abscesses, including one with a concomitant prostatic and liver abscesses. Interestingly, none of the patients presented with appendicitis despite it being one of the two primary aetiologies of pylephlebitis, alongside diverticulitis [3,4,5,6,7]. This could be attributable to our hospital’s profile: a monodisciplinary infectious diseases hospital without surgical services; thus, the appendicitis cases are referred directly to surgical departments. Another particularity of our cases is that nearly all patients exhibited abscesses, which could be either a source, as in the cases presented with prostatic or splenic abscess, or rather a consequence of pylephlebitis, as in the cases of liver abscess. This high prevalence of abscesses is consistent with our institution’s monodisciplinary focus on infectious diseases, where patients were addressed relatively late in the disease’s evolution, allowing time for abscess formation. An additional explanation for the predominance of liver abscesses will be explored further in Section 4.2.

### 4.2. Clinical Presentation in Pylephlebitis 

PVT clinical presentation depends on whether the thrombosis is acute or chronic, its cause, and the underlying pathology. In the case of pylephlebitis, the event is rather acute, characterised by acute PVT, meaning with a duration of less than six months [30]. This may coexist with preexisting comorbidities that, independently, can predispose to non-infectious PVT (whether acute or chronic), resulting in a clinical presentation that varies in duration, signs, and symptoms.

The most common symptoms at admission are fever (53–75.5%) and abdominal pain (66.4–91%). The abdominal pain character: variable intermittent or colicky pain especially in the context of an underlying intra-abdominal infection or inflammatory process could suggest a portal thrombosis, even an extension to mesenteric vein thrombosis and a consecutive mesenteric ischemia [5]. Other symptoms include diarrhoea, haematochezia, vomiting, and nausea [4,5]. On physical examination, the abdomen may be distended and show signs of peritoneal irritation (in cases of abdominal inflammation, intestinal perforation, or mesenteric infarction) or the absence of intestinal sounds in cases of ileus. In non-cirrhotic patients, even if ascites is present, it is in small amounts and detectable only with ultrasound or other imaging exams. If clinical examination reveals ascites in non-cirrhotic patients, clinicians should consider bowel ischemia and intestinal infarction [31,32].

All our patients presented with fever, and half of them had abdominal pain; the pain was either localised to the upper abdomen—specifically the epigastric region and right hypochondrium or diffuse. In two patients, it was described as intermittent or colicky in nature. Other symptoms, such as nausea/vomiting, weight loss, diarrhoea, and jaundice, were less common. In two cases, patients experienced confusion and loss of consciousness, signs of severe systemic complications.

Liver abscesses were described in 2% to 8.5% of pylephlebitis cases [4,5] and are considered either a cause or a complication of infective PVT, when portal thrombosis extends to intrahepatic portal branches, or a complication of the primary infection, which spreads by contiguity, such as in diverticulitis or pancreatitis [3,4,33]. Patients with hepatic abscesses could be less symptomatic but often exhibit signs such as unexplained weight loss [34], right upper quadrant pain, jaundice, fever, and chills [35]. Jaundice and elevated transaminases are not common; most patients with hepatic abscesses have elevated alkaline phosphatase (especially those abscesses resulting from contiguity) [36].

In our study group, hepatic abscesses were the main finding on abdominal imaging. At least one liver abscess was detected in 12 out of 15 (80%) cases, a rate significantly higher than that reported by other authors. As discussed in Section 4.1, the underlying explanation is likely multifactorial. A selection bias may be present, stemming from the specific profile of pylephlebitis cases admitted to our hospital. Patients presenting with acute symptoms dominated by abdominal pain are typically referred to surgical departments, whereas those with more insidious symptoms—primarily fever—are admitted to the infectious diseases ward. The latter group often experiences a longer disease course, allowing time for hepatic abscesses to develop. Conversely, patients with established liver abscesses require prolonged antibiotic therapy and are frequently referred to tertiary care infectious disease settings such as ours. Therefore, these findings cannot be extrapolated to the general population.

### 4.3. Paraclinical Findings in Pylephlebitis 

#### 4.3.1. Routine Laboratory Tests

Laboratory findings include inflammatory markers, such as leucocytosis, elevated C-Reactive Protein, and a high erythrocyte sedimentation rate [3,4,7,27]. In our series, inflammatory markers were commonly elevated, with a median CRP level approximately 20 times higher than normal and a high fibrinogen level. All patients with available procalcitonin measurements had elevated levels, with half of them exceeding five ng/mL, which is an indirect marker of sepsis.

Anaemia and hypoalbuminemia are commonly reported [7,27]. In our series, all but one patient had mild or moderate anaemia.

In contrast to non-infectious PVT, liver function in pylephlebitis is usually preserved, except in individuals with pre-existing liver disease [2]. Transaminase levels may be elevated in most cases, accompanied by three- to fourfold increases in alkaline phosphatase or five- to tenfold increases in gamma-glutamyl transferase in up to 40% of patients. Hyperbilirubinemia can reach high levels and occurs in over 55% of patients [7,27]. In our study group, both ALT and AST were slightly elevated in two-thirds of cases. GGT was elevated in all cases, with a median value twice the upper limit of normal, while bilirubin levels were normal in most cases or only very mildly elevated.

Although D-dimer levels were not systematically assessed across the entire cohort, they were markedly elevated in all patients for whom measurements were available. This observation is consistent with previous reports, where elevated D-dimer has been frequently associated with pylephlebitis [4]. More broadly, D-dimer elevation in the context of intra-abdominal infection may suggest an underlying pylephlebitis and should prompt systematic evaluation—including appropriate imaging studies—to confirm the presence of thrombosis. Early identification is crucial, as the addition of anticoagulant therapy in confirmed cases may contribute to improved clinical outcomes.

#### 4.3.2. Etiological Agent(s)

Due to the monodisciplinary profile of the hospital, which is a tertiary care infectious diseases centre with no surgical facilities, the gold standard method for diagnosing the aetiological agent of pylephlebitis—direct cultures of the thrombotic material obtained through aspiration of the portal vein—was not feasible. Even in modern settings, with surgical facilities, this diagnostic approach remains challenging to implement, and imaging examinations that identify an intra-abdominal source of infection should be sufficient for diagnosis. Since direct cultures from thrombotic material are rarely performed, it is highly recommended to obtain blood cultures in patients with a strong suspicion of pylephlebitis, which can be positive in up to 70% [4].

Regarding microbiology, pylephlebitis can be caused by one or more agents [4,5,7,27,37]. The most frequently identified bacteria are *Escherichia coli*, *Bacteroides* spp., and *Streptococcus* spp., which account for 25%, 17%, and 15% of cases, respectively, in the largest systematic review published in 2023, analysing 220 pylephlebitis cases [4], consistent with another review published in 2022, by Jevtic et al., which included 103 patients: *E. coli*, *Bacteroides* spp., *Streptococcus* spp., and *Fusobacterium* spp. (accounting for 20.4%, 12.6%, 11.7% and 9.7%, respectively) [7]. Some studies report that *Bacteroides fragilis* is the most common germ isolated in monobacterial infections [27]. Invasive *Bacteroides fragilis* infection can cause thrombotic phenomena through several mechanisms, such as heparinase production, fibrin clotting, and macrophage activation by a cell-wall component [38]. Other mechanisms, such as the transient production of anti-cardiolipin antibodies, have been proposed by different authors [39]. *Klebsiella pneumoniae* is not listed among the primary aetiological agents of pylephlebitis, despite being a leading pathogen in hepatic abscesses [40,41]. This discrepancy is likely due to the low proportion of liver abscesses reported in published case series of pylephlebitis. In our series of 15 patients, 80% of whom presented with liver abscesses, *Klebsiella pneumoniae* was identified in half of the cases with positive blood cultures, underscoring its prominence.

Most cases of hepatic abscesses caused by *Klebsiella pneumoniae* are associated with hypervirulent strains that exhibit a hypermucoviscous phenotype and belong to the capsular serotypes K1 and K2 [42]. This phenotype is characterised by longer capsular polysaccharide chains and resistance to clearance by Kupffer cells in animal models, which explains its ability to produce multiple liver abscesses and metastatic infections [43]. Portal vein thrombophlebitis associated with a hypervirulent *K. pneumoniae* strain was recently reported [44]. In our series, hypervirulent *K pneumoniae* strain was not documented.

#### 4.3.3. Imaging in Pylephlebitis

Ultrasound has a sensitivity of 60–100% for diagnosing PVT. It detects a mass in the portal vein lumen or within its branches: in acute PVT, the material is hypo- or isoechoic, whereas in chronic PVT, it is hyperechoic. Doppler examination shows alterations in portal flow, such as reduced portal velocity, flow defects and dilation or absent compressibility of the portal venous system [5,45]. Doppler examination may also reveal multiple tortuous small vessels replacing the portal vein, which is compatible with a diagnosis of cavernoma [5,29].

Abdominal CT with intravenous contrast is the most effective diagnostic tool for diagnosing pylephlebitis. Abdominal CT has significant advantages over ultrasonography, not just because it may detect an underlying cause but also could detect thrombus extension into the mesenteric circulation and identify potential PVT complications such as hepatic abscesses, bowel ischemia and/or perforation, and chronic thrombosis [3,5,31,46,47]. Abdominal MRI is also helpful for PVT diagnosis, with a sensitivity of 100% for PVT detection and a specificity of approximately 98% [48].

Re-evaluation with imaging is recommended seven days after starting antibiotic therapy to monitor thrombus extension or other complications [27] and then, to be repeated every three months until the clot regresses [30].

We did not find significant differences in the portal venous branches affected by thrombosis, with the left, right, and main portal veins being equally involved: five, four, and five cases, respectively. However, only portal vein thrombosis was described in the other six cases, with no further details provided. Another finding includes two cases of thrombosis extension involving the suprahepatic veins and a cavernoma in the hepatic hilum, resulting from chronic tortuous transformation of the left branch of the portal vein.

### 4.4. Treatment of Pylephlebitis

#### 4.4.1. Antibiotics

Antibiotic treatment is paramount. It involves antibiotics that target Gram-negative aerobes intestinal bacteria (such as *E. coli*, *Klebsiella* spp.), anaerobes (Gram-negative—*Bacteroides* spp., or Gram-positive *Fusobacterium* spp.), and Gram-positive bacteria (such as streptococci, enterococci). Commonly, a combination of metronidazole with either ceftriaxone, cefotaxime, ciprofloxacin, or levofloxacin is used, or monotherapy with piperacillin/tazobactam, ampicillin/sulbactam, or a carbapenem [49]. Antibiotic de-escalation is recommended based on culture results. The duration of antibiotic therapy lasts four weeks or more, extending up to six weeks in cases of liver abscesses [41].

Our findings indicate a strong preference for broad-spectrum regimens, particularly carbapenems, reflecting the severe nature of pylephlebitis and the predominant aetiology involving Gram-negative bacteria. Although our isolates were susceptible, the antibiotic treatment was rarely de-escalated.

#### 4.4.2. Anticoagulant and Recanalization (Thrombolytic) Treatment

Anticoagulation is the cornerstone of therapy in acute non-tumoral, non-cirrhotic PVT, as is pylephlebitis, and should be initiated at diagnosis—ideally in the first week [2,50,51]. Although there are no guidelines for anticoagulant therapy in pylephlebitis, as it is a rare condition, observational studies indicate that anticoagulants promote portal vein recanalization (minimising serious sequelae) and prevent the extension of thrombosis (which could lead to severe consequences).

The recommendations for anticoagulation in pylephlebitis are stronger if the occlusion is extended or in extension, if the mesenteric veins are affected (due to potential intestinal ischemia, necrosis. or perforation), They include the coexistence of conditions associated with hypercoagulability (haematological disease, neoplastic disease, etc.), mesenteric vein thrombosis or occlusion, and intestinal ischaemia and intractable sepsis [4,7,8,28,30].

Anticoagulant treatment can be initiated with heparin, maintained for 2–3 weeks, followed by oral anticoagulants, either VKA or DOACs. Recent studies [50] and an American Gastroenterology Association (AGA) Clinical Practice Update on PVT in cirrhosis 2025 [30] consider that VKA, DOACs and LMWH are all reasonable therapeutic options, including at the start of treatment.

If for the cirrhotic patients [30] or for those with procoagulant conditions (haematological conditions, such as thrombophilia, or malignancies), the duration of anticoagulant treatment is relatively clearly established (e.g., lifelong for those with procoagulant status), in non-cirrhotic patients, without procoagulant status, the optimal duration of anticoagulant therapy remains uncertain. The objective is to achieve recanalization of the portal vein, and since recanalization occurs within 4–6 months, most authors agree that anticoagulant therapy should be maintained for at least six months [2,4,17,52], and potentially longer, even lifelong if prothrombotic conditions are present.

Thrombolytic therapy, through direct trans-hepatic infusion into the portal vein or indirect injection into the superior mesenteric artery of thrombolytic drugs, is an option in very recent PVT [7,8] and may be considered in special circumstances.

#### 4.4.3. Surgery in Pylephlebitis

Surgical treatment may be required to control the source of infection, especially in cases with intra-abdominal localised infections, such as appendicitis or cholecystitis, and to drain the liver abscesses in some circumstances. Bowel infarction may also require surgery.

In our series, four patients were referred for surgical intervention: three underwent procedures to drain liver abscesses or treat intra-abdominal abscesses secondary to diverticulitis, and one required cholecystectomy.

### 4.5. Evolution, Complications, and Prognosis

Sepsis and metastatic abscesses are the most common complications. Septic emboli may migrate from the suppurative thrombosed veins towards the liver, leading to hepatic abscesses [5,7,16,53,54] or disseminate hematogenous to the lungs and brain, resulting in abscess formation. Sepsis and multiple organ failure-sepsis-related could significantly increase (almost double) the fatality rate [16,17,55,56].

Mesenteric ischemia and subsequent bowel infarction are less common but much more severe, with in-hospital mortality ranging between 20% and 50% [57].

Portal hypertension represents a tardive consequence of pylephlebitis [17]. Related to this, a common complication is the persistence of thrombosis, which can lead to cavernous transformation of the portal vein [5,8,17,29], meaning the formation of new veins within or around the thrombotic segment. One case in our series was a 38-year-old male, known with preexisting thrombophilia, who was admitted for intra-abdominal sepsis, with positive blood culture for *B. fragilis*, had extended PVT, and presented a cavernous transformation of the left portal branch in the hepatic hilum, compatible with a cavernoma.

Infections with multiple pathogens may be associated with more severe progression according to some authors [58], while others suggest that polymicrobial infections do not increase the risk of mortality [7,59].

In the cohort of 103 cases, analysed by Jetvik et al. [7], positive blood cultures, comorbidities (such as cirrhosis, malignancies) and sepsis are considered independent risk factors for a poor prognosis, with increases in mortality of 2.2-fold, 5.5-fold and 17-fold, respectively. In fact, sepsis is by far the strongest predictor of fatality in pylephlebitis, underlying once more the importance of prompt and aggressive antibiotic treatment [7].

Other factors with prognostic significance include the location of the thrombus, the extent of portal occlusion, and the degree of extension into portal branches, the superior mesenteric vein, or the splenic vein [45].

In our series, two patients died because of severe sepsis and multi-organ failure. These findings are consistent with previous reports, where sepsis is identified as the leading cause of mortality in pylephlebitis [7,17].

### 4.6. The Study Limitations

This study has several limitations that need to be acknowledged. Firstly, the retrospective design spanning over two decades inevitably led to missing and incomplete data, heterogeneity in diagnostic approaches, including the use of computed tomography versus abdominal ultrasonography, and incomplete microbiological confirmation (all isolates were from blood cultures, and none from direct cultures from thrombotic material or intra-abdominal collections). Another consequence of the retrospective design is the absence of systematic follow-up information, which prevents assessment of long-term outcomes and late complications.

Secondly, pylephlebitis remains a rare condition, with only a limited number of cases reported in the literature, and our series of 15 patients is still too small to allow for robust statistical analysis or generalisable conclusions.

Finally, the lack of consensus in the literature and the absence of standardised guidelines on the management of pylephlebitis—especially regarding the role and duration of anticoagulation, the optimal antibiotic regimens and their duration, and the role and optimal timing for repeat imaging—limit our ability to compare our results with other published series and emphasise the need for future multicentre, prospective studies to address these issues.

## 5. Conclusions

Pylephlebitis is a rare complication of intra-abdominal infections. Maintaining a high level of suspicion can prevent unfavourable outcomes. Computed tomography is highly recommended when an intra-abdominal infection is suspected. Moreover, blood culture results may improve antibiotic management, enable de-escalation, and help prevent the emergence of resistance and adverse reactions. Broad-spectrum antibiotics targeting Gram-negative bacteria and anaerobes should be initiated before blood culture results are available. Anticoagulation therapy may improve outcomes and prevent severe progression and long-term sequelae. 

## Figures and Tables

**Table 1:** Demographics, Comorbidities, and Diagnosis of the Patients with Pylephlebitis.

No.	Sex Age	Comorbidities	Clinical	Intra- Abdominal Infection	Imagery (US/CT)	Blood Cultures
#1	M 24	HIV infection History of tuberculosis	Fever Sepsis Jaundice Profuse sweating Deterioration of general condition Weight loss	Undetermined	(CT): PVT Hepatosplenomegaly Multiple intra-abdominal enlarged lymph nodes	Negative
#2	M 66	Diabetes Alcohol abuse	Fever and chills Abdominal pain (epigastric) Myalgia and arthralgia Loss of appetite Physical asthenia	Liver abscess (multiple)	(US): Left branch PVT Multiple liver abscesses	Negative
#3	M 44	Burns of 2/3 degree	Fever	Liver abscess (multiple)	(CT): Multiple liver abscesses	Negative
#4	M 60	Diabetes	Low-grade fever and chills Diarrhoea Confusion Physical asthenia	Liver abscess (two)	(US): Main PVT (CT): Two liver abscesses Pancreatic head tumour without local invasion	*Klebsiella pneumoniae* *Escherichia coli*
#5	M 67	Cardiac pathology Aortic prosthesis History of Pyocholecystitis and liver abscesses	Fever	Cholecystitis Liver abscess (multiple)	(CT): Right branch PVT Multiple hepatic abscesses Cholecystitis (US): Portal vein gas Multiple hepatic abscesses	Negative
#6	F 58	Cardiac pathology Diabetes Alcohol abuse	Fever and chills Abdominal pain (intermittent, right hypochondrium) Nausea and vomiting	Liver abscess (not detailed)	(CT): PVT; Suprahepatic vein thrombosis Hepatic abscess	*Klebsiella* *pneumoniae*
#7	M 48	Cardiac pathology COPD Obesity Diverticulosis	Fever Abdominal pain (colicky, diffuse)	Diverticulitis Intraabdominal abscess (not detailed)	(CT): Main PVT Portal vein gas Diverticulitis with secondary abscess	*Klebsiella* *pneumoniae* *Enterococcus avium*
#8	M 75	Cardiac pathology Ictus Alcohol abuse	Fever	Splenic abscess Liver abscess (multiple)	(CT): Right branch PVT Hepatic and splenic abscesses	Negative
#9	F 64	Hypertension Diabetes Recent history of Pyocholecystitis	Fever	Cholecystitis Liver abscess (not detailed)	(CT): PVT Hepatosplenomegaly Hepatic abscess Cholecystitis	Negative
#10	M 83	Dementia Alcohol abuse	Fever and chills Abdominal pain (diffuse) Loss of appetite Loss of consciousness	Splenic abscess Prostatic abscess Liver abscess (multiloculated)	(CT): Left branch PVT Multiloculated hepatic abscess Splenic abscesses Prostatic abscess	*Bacteroides* *fragilis*
#11	M 16	No comorbidities	Fever Abdominal pain Dry cough Loss of appetite Deterioration of general condition	Splenic abscess Liver abscess (multiple)	(CT): PVT Multiple hepatic abscesses Dilatation of intrahepatic bile ducts (US): Multiple hepatic abscesses Multiple splenic abscesses	*Bacteroides* *fragilis* *Clostridium symbiosum*
#12	M 51	Hypertrophic cardiomyopathy	Fever Nausea Myalgia	Liver abscess (not detailed)	(CT): Main PVT; Suprahepatic vein thrombosis Hepatic abscess	*Klebsiella pneumoniae* *Pseudomonas aeruginosa*
#13	M 39	Alcohol abuse	Fever and chills Nausea Weight loss Abdominal pain (epigastric and right hypochondrium)	Liver abscess (multiple)	(CT): Main PVT; Porto-splenic confluence thrombosis Hepatosplenomegaly Multiple hepatic abscesses	Negative
#14	F 74	Cardiac pathology Antiphospholipid syndrome Diverticulosis	Fever Abdominal pain (diffuse) Diarrhoea	Liver abscess (not detailed)	(CT): PVT Hepatic abscess	*Escherichia coli*
#15	M 38	Thrombophilia HIV newly Dx Obesity	Fever; chills Abdominal pain (epigastric and right hypochondrium) Nausea; vomiting Diarrhoea	Diverticulitis Pancreatitis	(CT): Total PVT; Splenic and Superior Mesenteric veins thrombosis; Right branch PVT Portal cavernoma (left portal branch transformation) Splenic infarction	*Bacteroides* *fragilis*

Legend: Dx—diagnosis; COPD—chronic obstructive pulmonary disease; US—ultrasound (abdominal); CT—CT scan (abdominal); PVT—portal vein thrombosis.

**Table 2:** The treatment and the outcome of the patients diagnosed with pylephlebitis.

No	Sex Age	Antibiotic Therapy	ATB Duration (Days)	Anti Coagulation	Surgery	Outcome
#1	M, 24	Meropenem (20 days)	20	No	No	Death
#2	M, 66	Ciprofloxacin (7 days) → Levofloxacin + Ampicillin/Sulbactam (23 days)	30	LMWH	No	Favourable
#3	M, 44	Meropenem + Linezolid + Colistin (23 days) → Meropenem + Linezolid + Fluconazole (14 days) → Linezolid + Tigecycline (1 day) → Tigecycline (24 days)	62	LMWH VKA	No	Favourable
#4	M, 60	Tigecycline + Ceftazidime (25 days) → Tigecycline (10 days) → Moxifloxacin + Ceftazidime (6 days)	41	LMWH	No	Death
#5	M, 67	Piperacillin/Tazobactam + Gentamicin (14 days) → Piperacillin/Tazobactam (37 days)	51	VKA (started before diagnosis)	No	Favourable Fibrosis in the affected territory
#6	F, 58	Ampicillin/Sulbactam (5 days) → Piperacillin/Tazobactam (2 days) → Ertapenem + Teicoplanin (11 days)	42	LMWH	Yes (liver abscess drainage)	Unknown
#7	M, 48	Tigecycline + Meropenem + Linezolid + Colistin (21 days) → Tigecycline + Meropenem + Colistin (21 days)	18	LMWH VKA	Yes (drainage of intra-abdominal abscess)	Favourable
#8	M, 75	Meropenem + Vancomycin (14 days)	14	LMWH	No	Favourable
#9	F, 64	Meropenem + Vancomycin (5 days) → Meropenem (21 days)	26	LMWH	Yes (Cholecystectomy)	Favourable
#10	M, 83	Ertapenem (25 days) → Tigecycline (9 days) → Ceftriaxone + Metronidazole (7 days) → Metronidazole (7 days)	23	LMWH	No	Favourable
#11	M, 16	Ceftriaxone (2 days) → Cefotaxime (6 days) → Ciprofloxacin + Sulfamethoxazole/Trimethoprim (5 days) → Piperacillin/Tazobactam + Metronidazole (25 days) → Piperacillin/Tazobactam (8 days)	46	LMWH	No	Favourable Hepatic left lobe atrophy
#12	M, 51	Cefotaxime + Metronidazole (9 days) → Cefixime + Metronidazole (7 days) → Meropenem (22 days)	38	LMWH	No	Favourable
#13	M, 39	Piperacillin/Tazobactam (29 days)	29	LMWH	No	Favourable
#14	F, 74	Ampicillin/Sulbactam (7 days) → Ampicillin/Sulbactam (4 days) → Ertapenem (1 day)	12	VKA	Yes (drainage of hepatic abscess)	Unknown
#15	M, 38	Piperacillin/Tazobactam (6 days) → Metronidazole (10 days)	16	LMWH DOAC	No	Favourable Portal (hepatic hilus) cavernoma

Legend: LMWH—low-molecular-weight heparin; VKA—vitamin K antagonists; DOACs—direct oral anticoagulants. N.B.: Table 2 is a continuation of Table 1.

## Data Availability

The data presented in this article are available upon request from the corresponding authors. The raw patients’ data are stored in the electronic database of the National Institute for Infectious Diseases Matei Bals, Bucharest, Romania, and consist of their files (diagnosis, history, outcome, and all laboratory tests). Our Institute database has contained patients’ data since 2000.

## References

[1] Bayraktar Y., Harmanci O. (2006). Etiology and consequences of thrombosis in abdominal vessels. World J. Gastroenterol..

[2] Ponziani F.R., Zocco M.A., Campanale C., Rinninella E., Tortora A., Di Maurizio L., Bombardieri G., De Cristofaro R., De Gaetano A.M., Landolfi R. (2010). Portal vein thrombosis: Insight into physiopathology, diagnosis, and treatment. World J. Gastroenterol..

[3] Hartpence J., Woolf A. Pylephlebitis. [Updated 2023 Aug 13]. In: StatPearls [Internet]. Treasure Island (FL): StatPearls Publishing. https://www.ncbi.nlm.nih.gov/books/NBK563246/.

[4] Fusaro L., Di Bella S., Martingano P., Saveria Crocè L.S., Giuffrè M. (2023). Pylephlebitis: A systematic review. Diagnostics.

[5] Choudhry A.J., Baghdadi Y.M., Amr M.A., Alzghari M.J., Jenkins D.H., Zielinski M.D. (2016). Pylephlebitis: A review of 95 cases. J. Gastrointest. Surg..

[6] Belhassen-García M., Gomez-Munuera M., Pardo-Lledias J., Velasco-Tirado V., Perez-Persona E., Galindo-Perez I., Alvela-Suárez L., Romero-Alegría Á., Muñoz-Bellvis L., Cordero-Sánchez M. (2014). Pylephlebitis: Incidence and prognosis in a tertiary hospital. Enferm. Infecc. Microbiol. Clin..

[7] Jevtic D., Gavrancic T., Pantic I., Nordin T., Nordstrom C.W., Antic M., Pantic N., Kaljevic M., Joksimovic B., Jovanovic M. (2022). Suppurative Thrombosis of the Portal Vein (Pylephlebitis): A Systematic Review of Literature. J. Clin. Med..

[8] Chawla Y.K., Bodh V. (2015). Portal Vein Thrombosis. J. Clin. Exp. Hepatol..

[9] Intagliata N.M., Caldwell S.H., Tripodi A. (2019). Diagnosis, development, and treatment of portal vein thrombosis in patients with and without cirrhosis. Gastroenterology.

[10] Condat B., Valla D. (2006). Nonmalignant portal vein thrombosis in adults. Nat. Clin. Pract. Gastroenterol. Hepatol..

[11] Poultsides G.A., Lewis W.C., Feld R., Walters D.L., Cherry D.A., Ruby S.T. (2005). Portal vein thrombosis after laparoscopic colectomy: Thrombolytic therapy via the superior mesenteric vein. Am. Surg..

[12] Bick R.L. (1992). Coagulation abnormalities in malignancy: A review. Semin. Thromb. Hemost..

[13] Tripodi A., Branchi A., Chantarangkul V., Clerici M., Merati G., Artoni A., Mannucci P.M. (2011). Hypercoagulability in patients with type 2 diabetes mellitus detected by a thrombin generation assay. J. Thromb. Thrombolysis.

[14] Bucciarelli P., Martinelli I., Artoni A., Passamonti S.M., Previtali E., Merati G., Tripodi A., Mannucci P.M. (2012). Circulating microparticles and risk of venous thromboembolism. Thromb. Res..

[15] Van Montfoort M.L., Stephan F., Lauw M.N., Hutten B.A., Van Mierlo G.J., Solati S., Middeldorp S., Meijers J.C., Zeerleder S. (2013). Circulating nucleosomes and neutrophil activation as risk factors for deep vein thrombosis. Arterioscler. Thromb. Vasc. Biol..

[16] Kumar V., Abbas A.K., Aster J.C. (2014). Robbins and Cotran Pathologic Basis of Disease.

[17] Basit S.A., Stone C.D., Gish R.G. (2015). Portal Vein Thrombosis. Clin. Liver Dis..

[18] Chaudhary H., Jindal P., Pandiarajan V., Kumar J., Sudhakar M., Ezhumalai G., Nada R., Gupta K. (2021). Portal vein thrombosis, livedo reticularis, polymicrobial sepsis and high antiphospholipid antibody titers in a newborn: A clinicopathological conference of antiphospholipid-associated neonatal syndrome. Lupus.

[19] Baril N., Wren S., Radin R., Ralls P., Stain S. (1996). The role of anticoagulation in pylephlebitis. Am. J. Surg..

[20] Tursi A. (2010). Diverticular disease: A therapeutic overview. World J. Gastrointest. Pharmacol. Ther..

[21] Pradka S.P., Trankiem C.T., Ricotta J.J. (2012). Pylephlebitis and acute mesenteric ischemia in a young man with inherited thrombophilia and suspected foodborne illness. J. Vasc. Surg..

[22] Calomino N., Carbone L., Kelmendi E., Piccioni S.A., Poto G.E., Bagnacci G., Resca L., Guarracino A., Tripodi S., Barbato B. (2025). Western Experience of Hepatolithiasis: Clinical Insights from a Case Series in a Tertiary Center. Medicina.

[23] Chau N., Bhatia S., Raman M. (2007). Pylephlebitis and pyogenic liver abscesses: A complication of hemorrhoidal banding. Can. J. Gastroenterol. Hepatol..

[24] De Roover A., Detry O., Coimbra C., Hamoir E., Honoré P., Meurisse M. (2006). Pylephlebitis of the portal vein complicating intragastric migration of an adjustable gastric band. Obes. Surg..

[25] Tandon R., Davidoff A., Worthington M.G., Ross J.J. (2005). Pylephlebitis after CT-guided percutaneous liver biopsy. Am. J. Roentgenol..

[26] James A.W., Rabl C., Westphalen A.C., Fogarty P.F., Posselt A.M., Campos G.M. (2009). Portomesenteric venous thrombosis after laparoscopic surgery: A systematic literature review. Arch. Surg..

[27] Kanellopoulou T., Alexopoulou A., Theodossiades G., Koskinas J., Archimandritis A.J. (2010). Pylephlebitis: An overview of non-cirrhotic cases and factors related to outcome. Scand. J. Infect. Dis..

[28] Naymagon L., Tremblay D., Schiano T., Mascarenhas J. (2020). The role of anticoagulation in pylephlebitis: A retrospective examination of characteristics and outcomes. J. Thromb. Thrombolysis.

[29] De Gaetano A.M., Lafortune M., Patriquin H., De Franco A., Aubin B., Paradis K. (1995). Cavernous transformation of the portal vein: Patterns of intrahepatic and splanchnic collateral circulation detected with Doppler sonography. AJR Am. J. Roentgenol..

[30] Davis J.P.E., Lim J.K., Francis F.F., Ahn J. (2025). AGA Clinical Practice Update on Management of Portal Vein Thrombosis in Patients With Cirrhosis: Expert Review. Gastroenterology.

[31] Kocher G., Himmelmann A. (2005). Portal vein thrombosis (PVT): A study of 20 non-cirrhotic cases. Swiss Med. Wkly..

[32] Elkrief L., Corcos O., Bruno O., Larroque B., Rautou P., Zekrini K., Bretagnol F., Joly F., Francoz C., Bondjemah V. (2014). Type 2 diabetes mellitus as a risk factor for intestinal resection in patients with superior mesenteric vein thrombosis. Liver Int..

[33] Al-Hamid H., Manatsathit W., Johnson L., Barawi M. (2014). Pylephlebitis with pyogenic liver abscesses: A rare complication of pancreatitis. Am. J. Gastroenterol..

[34] Wireko M., Berry P.A., Brennan J., Aga R. (2005). Unrecognized pylephlebitis causing life-threatening septic shock: A case report. World J. Gastroenterol..

[35] Boiko V.V., Tishchenko A.M., Gusak I.V., Maloshtan A.A., Skoryi D.I., Smachilo R.M. (2013). Surgical treatment of a solitary hepatic abscess. Klin. Kir..

[36] Huang C.J., Pitt H.A., Lipsett P.A., Osterman F.A., Lillemoe K.D., Cameron J.L., Zuidema G.D. (1996). Pyogenic hepatic abscess. Changing trends over 42 years. Ann. Surg..

[37] Anand S., Umeh C.A., Giberson C., Wassel E., Nguyen A., Porter H., Choday P., Kaur H., Kundu A., Penaherrera J. (2021). Septic Portal Vein Thrombosis, Clinical Presentation, and Management. Cureus.

[38] Rodrigues C., Siciliano R.F., Zeigler R., Strabelli T.M. (2012). Bacteroides fragilis endocarditis: A case report and review of literature. Braz. J. Infect. Dis..

[39] Liappis A.P., Roberts A.D., Schwartz A.M., Simon G.L. (2003). Thrombosis and infection: A case of transient anti-cardiolipin antibody associated with pylephlebitis. Am. J. Med. Sci..

[40] Abbas M.T., Khan F.Y., Muhsin S.A., Al-Dehwe B., Abukamar M., Elzouki A.N. (2014). Epidemiology, Clinical Features and Outcome of Liver Abscess: A single Reference Center Experience in Qatar. Oman Med. J..

[41] Akhondi H., Sabih D.E. Liver Abscess. [Updated 2023 Jul 3]. In: StatPearls [Internet]. Treasure Island (FL): StatPearls Publishing. https://www.ncbi.nlm.nih.gov/books/NBK538230/.

[42] Popescu G.A., Tanase D., Petrescu A.M., Florea D. (2014). Liver abscess associated with severe myopathy caused by Klebsiella pneumoniae serotype K1 in Romania. J. Infect. Dev. Ctries..

[43] Angeles-Solano M., Tabashsum Z., Chen L., Rowe S.E. (2025). Klebsiella pneumoniae liver abscesses: Pathogenesis, treatment, and ongoing challenges. Infect. Immun..

[44] Kalangi H., Yancovitz S.R., Camins B. (2024). A unique case of hypervirulent Klebsiella pneumoniae acute cholecystitis complicated by portal vein thrombophlebitis: A case report and literature review. IDCases.

[45] Sarin S.K., Philips C.A., Kamath P.S., Choudhury A., Maruyama H., Nery F.G., Valla D.C. (2016). Toward a comprehensive new classification of portal vein thrombosis in patients with cirrhosis. Gastroenterology.

[46] Balthazar E.J., Gollapudi P. (2000). Septic Thrombophlebitis of the Mesenteric and Portal Veins: CT Imaging. J. Comput. Assist. Tomogr..

[47] Webster G.J., Burroughs A.K., Riordan S.M. (2005). Review article: Portal vein thrombosis—New insights into aetiology and management. Aliment. Pharmacol. Ther..

[48] Shah T.U., Semelka R.C., Voultsinos V., Elias J., Altun E., Pamuklar E., Firat Z., Gerber D.A., Fair J., Russo M.W. (2006). Accuracy of magnetic resonance imaging for preoperative detection of portal vein thrombosis in liver transplant candidates. Liver Transpl.

[49] Sanford Guide to Antimicrobial Therapy 2025 App Version 7.0.9. https://www.sanfordguide.com/.

[50] Nery F., Valadares D., Morais S., Gomes M.T., De Gottardi A. (2017). Efficacy and safety of direct-acting oral anticoagulants use in acute portal vein thrombosis unrelated to cirrhosis. Gastroenterol. Res..

[51] Janczak D.T., Mimier M.K., McBane R.D., Kamath P.S., Simmons B.S., Bott-Kitslaar D.M., Lenz C.J., Vargas E.R., Hodge D.O., Wysokinski W.E. (2018). Rivaroxaban and apixaban for initial treatment of acute venous thromboembolism of atypical location. Mayo Clin. Proc..

[52] Hale G.R., Sakkal L.A., Galanis T. (2019). Pylephlebitis treated with apixaban. Hosp. Pract..

[53] Chang Y.S., Min S.Y., Joo S.H., Lee S.H. (2008). Septic thrombophlebitis of the porto-mesenteric veins as a complication of acute appendicitis. World J. Gastroenterol..

[54] Pérez-Bru S., Nofuentes-Riera C., García-Marín A., Luri-Prieto P., Morales-Calderón M., García-García S. (2015). Pylephlebitis: A rare but possible complication of intra-abdominal infections. Cirugía Cir..

[55] Saxena R., Adolph M., Ziegler J.R., Murphy W., Rutecki G.W. (1996). Pylephlebitis: A case report and review of outcome in the antibiotic era. Am. J. Gastroenterol..

[56] Nigussie B., Woredekal D., Abaleka F.I., Gizaw M., Tharu B. (2020). A Sporadic Case of Disseminated Fusobacterium Causing Pylephlebitis and Intracranial and Hepatic Abscesses in a Healthy Young Patient. Cureus.

[57] Kumar S., Sarr M.G., Kamath P.S. (2001). Mesenteric venous thrombosis. N. Engl. J. Med..

[58] Murray J.L., Connell J.L., Stacy A., Turner K.H., Whiteley M. (2014). Mechanisms of synergy in polymicrobial infections. J. Microbiol..

[59] Shah P.M., Edwards B.L., Dietch Z.C., Guidry C.A., Davies S.W., Hennessy S.A., Duane T.M., O’Neill P.J., Coimbra R., Cook C.H. (2016). Do Polymicrobial Intra-Abdominal Infections Have Worse Outcomes than Monomicrobial Intra-Abdominal Infections?. Surg. Infect..

